# First person – Marion Lechable and Alexandre Jan

**DOI:** 10.1242/bio.057034

**Published:** 2020-11-05

## Abstract

First Person is a series of interviews with the first authors of a selection of papers published in Biology Open, helping early-career researchers promote themselves alongside their papers. Marion Lechable and Alexandre Jan are co-first authors on ‘An improved whole life cycle culture protocol for the hydrozoan genetic model *Clytia hemisphaerica*’, published in BiO. Marion is a Marie-Curie Sklodowska PhD student (ARDRE doctoral programme) in the lab of Professor Bert Hobmayer at University of Innsbruck, Institute of Zoology, Austria, investigating stem cell biology, regeneration, aging and cnidarians models. Alexandre was an engineer assistant in the lab of Dr Tsuyoshi Momose at Laboratoire de Biologie du Développement, at the Institut de la Mer de Villefranche, France, investigating capture breeding, cnidarians models, gelatinous plankton, improving culturing protocols and regeneration.


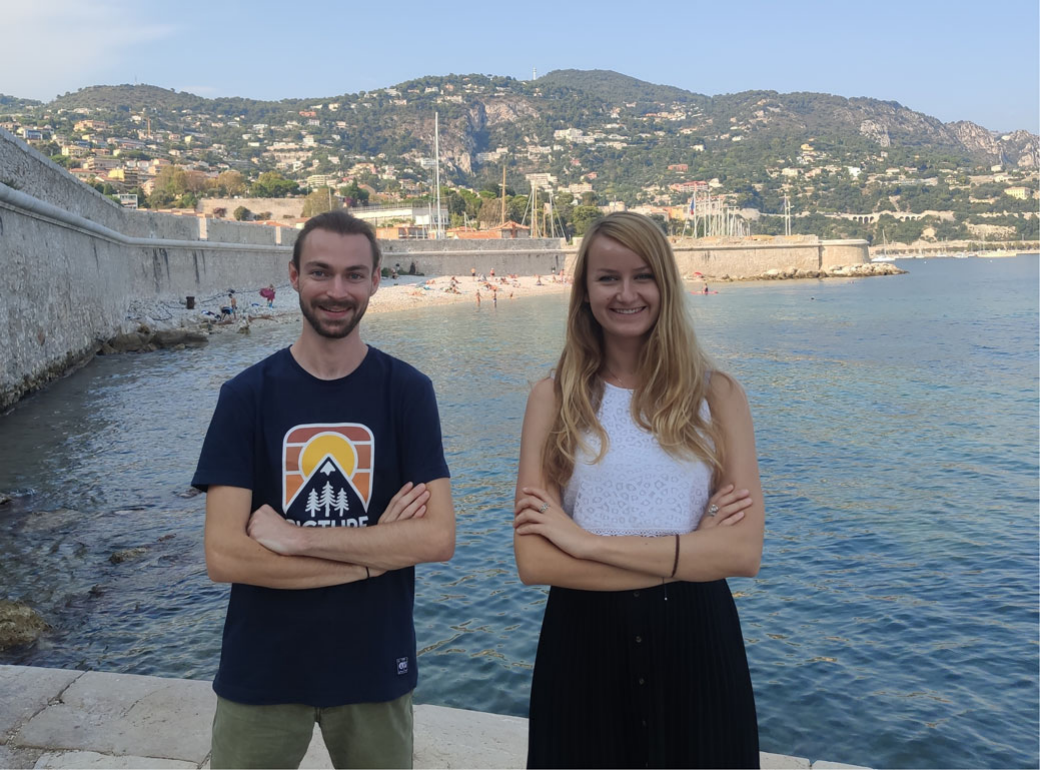


**Marion Lechable and Alexandre Jan**

**What is your scientific background and the general focus of your lab?**

**ML:** I did biotechnology studies and then I specialized in marine biology. I became rapidly interested in cnidarians, a metazoan group that includes corals and jellyfish. I was completely fascinated that some species from this clade possess strong regeneration abilities, do not undergo aging and could be considered as ‘quasi-immortal’. I first worked on the stem cell dynamics of the hydrozoan *Clytia hemisphaerica* in the developmental biology laboratory of Villefranche-sur-mer as an engineer assistant. Currently, I am a PhD student at the Institute of Zoology in Innsbruck, and I am working on *Hydra* within the Aging, Regeneration and Drug Research (ARDRE) European consortium.

**AJ:** I did a bachelor in aquaculture, finalized by an 8-month internship and followed by a short-term contract at the aquarium of the Oceanographic Museum of Monaco. There I had the opportunity to breed a huge diversity of marine species, from sea-horses to crustaceans, cephalopods, fish and jellyfish. Afterwards I joined the Marine Center Resources Service of the Marine Institute of Villefranche as an aquarist, and bred *Clytia hemisphaerica* jellyfish for the Development Biology Laboratory. Currently, I am an engineer assistant in the same institute. I breed several jellyfish species for genomic and regeneration research projects. I also work on improving culture protocols and establishing new gelatinous plankton species as research models.

**How would you explain the main findings of your paper to non-scientific family and friends?**

**ML:** Most biological research is conducted on classic organism models, such as mice or flies. They have already provided lots of discoveries, notably in the medical area. Since scientists are working with these standard model animals, they are very experienced in handling and maintaining them in their labs. However, this is not the case for more unconventional model organisms, which makes it difficult for scientists to explore the broad possibilities they are offering. Our working group has taken on the challenge to explore working with the non-standard model *Clytia* in the lab, which is a *Clytia* that belongs to a group of animals called Cnidaria, which diverged from other animals early during animal evolution, and thus could provide new insights into how higher animals, like humans, have acquired their diversity. In this scientific publication, we share the knowledge we gained during the last years working with *Clytia*. This will to enable other scientists to benefit from our experience with this organism.

**AJ:** *Clytia* is an unusual species, and as a research model, its easy cultivation opens new doors for many new research topics.

“*Clytia* is an unusual species, and as a research model, its easy cultivation opens new doors for many new research topics.”

**What are the potential implications of these results for your field of research?**

**ML:** We hope that this paper will be useful for all researchers in the field, who want to start studying *Clytia* or improve their culture, especially the mutant strains, and will help with extending the development of *Clytia* as a genetic model.

**AJ:** In addition to Marion’s remarks, I hope our article will help to establish other cnidarian species as tractable models.

**What, in your opinion, are some of the greatest achievements in your field and how has this influenced your research?**

**ML:** I think that the cnidarian community is becoming increasingly wide, with a great variety of species that have been developed as experimental models. Each species has a very specific lifecycle with different lifespans, stages and types of reproduction. Some of them do not even have jellyfish or polyp stages, probably due to a loss through evolution. Thus, developing a model such as *Clytia*, which have polyp and jellyfish stages, but are also able to do both sexual and asexual reproduction, was challenging. Altogether, with the molecular tools and resources available now, improving the culture system was the next step for this rising genetic model.”

**Figure:**
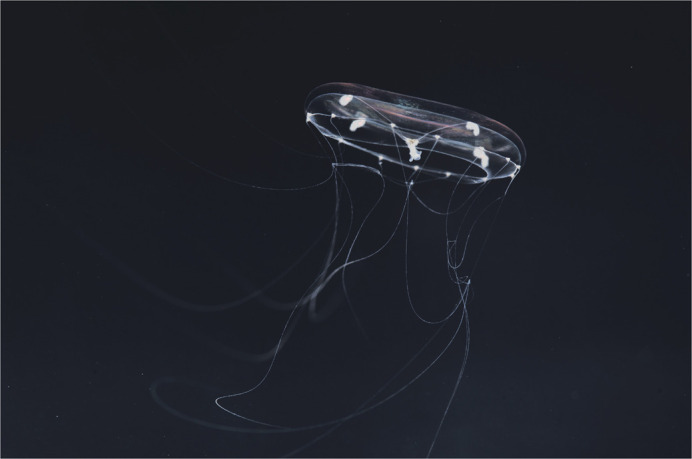
***Clytia hemisphaerica*. Photograph by Alexandre Jan and Marion Lechable.**

“… the cnidarian community is becoming increasingly wide, with a great variety of species that have been developed as experimental models.”

**What changes do you think could improve the professional lives of early-career scientists?**

**ML:** Being an early-career scientist is not something easy nowadays. We must stand out to succeed and get off the beaten tracks. I think as young researchers we should think beyond our own fields and harness several scientific domains. In my opinion, we should be prepared to do this earlier during university in order to improve our future professional lives.

**What's next for you?**

**ML:** As I am starting my first year of PhD, I aim to conduct my new project in Innsbruck (Austria). I am now working on another hydrozoan, *Hydra*, and I am trying to decipher stem cell decision making at the cellular and molecular levels, which may help us to understand regeneration processes and stem cell behavior. I am particularly interested into two core regulators, the Wnt/β-Catenin pathway and the oncogenic Myc transcription factor. Myc plays also a role in the mTOR pathway, involved in aging. I aim at bringing new insights by fully characterizing this gene in a highly regenerative species that does not age and never develops tumors.

**AJ:** I am still raising *Clytia* in Villefranche for several regeneration and genetics projects. I am also developing a culture system for the scyphozoan *Pelagia noctiluca,* with the aim of developing this species as a new genetic model.
